# Mental health and internet addiction: prevalence and risk factors among vocational high school students in Henan, China

**DOI:** 10.3389/fpsyg.2025.1555954

**Published:** 2025-09-24

**Authors:** Xiaorui Fan, Zhenti Cui, Rongrong Guan, Chuyi Zhan, Xiaofei Tian

**Affiliations:** ^1^School of Nursing, Fudan University, Shanghai, China; ^2^School of Medicine, Sias University, Zhengzhou, China; ^3^School of Nursing, Xi'an Jiaotong University, Shanxi, China; ^4^School of Art Design, Zhengzhou University of Industrial Technology, Zhengzhou, China; ^5^The Seventh Affiliated Hospital of Sun Yat-sen University, Shenzhen, China

**Keywords:** adolescents, internet addiction, mental health problems, risk factors, vocational high school students, China

## Abstract

**Objective:**

This study investigates the prevalence and influencing factors of mental health problems among vocational high school students in Henan Province, China, with varying levels of internet addiction.

**Methods:**

A cross-sectional survey was conducted between June and August 2018, involving 3,051 vocational high school students selected through stratified cluster sampling. Data were collected using demographic questionnaires, the Internet Addiction Test, and the Mental Health Inventory for Middle-School Students. Univariate and multivariate logistic regression analyses were applied to identify influencing factors.

**Results:**

The prevalence of mental health problems was 59% (95% CI: 57–61%). Among average, problematic, and addictive internet users, prevalence rates were 65% (95% CI: 63–67%), 54% (95% CI: 51–57%), and 34% (95% CI, 28–40%), respectively. Across the overall population and subgroups, physical illness, chronic constipation, and loneliness were consistently associated with higher mental health risks (*p* < 0.05). Additionally, ethnic minority status, sexual behavior, and lower maternal education were significant risk factors in the overall population, average users, and problematic users (*p* < 0.05). Smoking was also linked to higher mental health risks in the overall and average user groups (*p* < 0.05).

**Conclusion:**

Mental health issues among vocational high school students, particularly those with internet addiction, are prevalent and severe. Early interventions targeting key risk factors are critical to improving mental health outcomes in this population.

## 1 Introduction

Adolescence is a critical period of growth and development characterized by significant physical, cognitive, emotional, and social changes. Notably, 50% of adult mental health disorders originate before the age of 16, making adolescents particularly vulnerable to conditions such as depression and other mental health challenge (Patel et al., 2008; Vyas et al., 2015). According to the World Health Organization, mental health is a state of well-being where individuals can realize their potential, cope with normal life stresses, work productively, and contribute to their communities (Levav and Rutz, 2002). Adolescents with good mental health not only demonstrate vitality and social adaptability but also effectively utilize their mental and physical potential, showcasing positive psychological and social function (Levav and Rutz, 2002).

Global studies estimate that 10–20% of adolescents experience mental health problems (Waid and Kelly, 2020). Around one-fifth of these issues persist into adulthood, with the associated costs being ten times higher than those of adult-onset mental health disorders (Kessler et al., 2005). The World Health Organization projected that by 2020, mental health conditions in children would become a leading cause of illness, death, and disability globally (Membride, 2016). One study shows that mental health problems are highly prevalent among adolescents in rural China, with approximately 20% at risk for depression and 68% at risk for any type of anxiety, including learning anxiety (Jiang et al., 2022). These figures are significantly higher than the 10–20% worldwide average (Kieling et al., 2011).

Adolescents’ mental health issues often manifest as depression, anxiety, autism, attention-deficit/hyperactivity disorder (ADHD), and other psychological disorders. Behavioral problems, such as aggression or withdrawal, are also common (Schulte-Körne, 2016). These mental health challenges significantly impact adolescents’ social and educational development and increase their vulnerability to suicidal thoughts, behaviors, and even mortality (Patel et al., 2008). The factors contributing to adolescent mental health problems are multifaceted, encompassing personal and familial influences. For instance, overweight and obese adolescents are more prone to low self-esteem and body image anxiety, leading to heightened psychological stress (Myers and Rosen, 1999). Recent systematic reviews have linked internet addiction to late-night habits, impaired parent–child and school relationships, low mood, stress, anxiety, and behavioral problems, particularly ADHD, conduct disorders, and emotional symptoms (Sahu et al., 2019). Additional risk factors include smoking (Burki, 2016), alcohol abuse (Ning et al., 2020) and drug addiction (Pompili et al., 2012).

Regarding family factors, a large-scale cross-sectional study in China identified family closeness, conflicts, and parent–child separation as significant risk factors for mental health problems among middle and high school students (Xing et al., 2010). Similarly, a study of 988 Colombian adolescents revealed that family economic status, functionality, and structure were strongly associated with mental health outcomes (Apsley and Padilla-Walker, 2020). Supporting these findings, a UK cohort study involving 11,564 children aged 14 found that those with parents experiencing persistent poor mental health and poverty faced a heightened risk of developing socio-emotional and behavioral problems (Adjei et al., 2022). The widespread use of the internet, particularly social media, has fundamentally transformed how adolescents communicate, socialize, and maintain friendships. However, internet addiction—characterized by an uncontrollable urge to use the internet, often leading to severe nervous tension and aggression—has emerged as a prevalent mental health issue among adolescents. Globally, the prevalence of internet addiction among teenagers ranges from 1.98 to 35.8% (Pan et al., 2020). A total of 164 studies involving 737,384 adolescents were included in the meta-analysis. The overall pooled IA prevalence was 10.3% (Zheng et al., 2022). Common symptoms include sadness, low mood, and a loss of interest in daily activities. Effective interventions may involve promoting physical activity, spending more time with friends, and fostering healthy social networks (Liu et al., 2024). One study involving 7,729 rural adolescents in eastern China did identify a positive association between internet addiction (measured using the Internet Addiction Test) and depression (measured using the Zung self-rating depression scale) (Wu et al., 2016). There was a significant association between adolescent video game time and poorer mental health. Each additional hour of playing video games also increased the chance of having moderate or above symptoms (Li et al., 2022). Some of these studies have identified a higher prevalence of adolescent mental health problems among those with higher amounts of video game usage (Wartberg et al., 2017), including more symptoms of anxiety and depression (Kuss and Griffiths, 2012), as well as higher levels of stress (Porter and Goolkasian, 2019). And using a sample of 3,694 participants (aged 10–19) from the 2018 to 2020 China Family Panel Survey (CFPS). Internet use time has a significant negative impact on mental health by crowding out sleep duration and parent-adolescent communication (Ma and Sheng, 2023).

According to the *Key Outcomes of China’s National Education Statistics 2021*, there were over 13 million students enrolled in secondary vocational schools, representing a substantial student population (National Education Statistics Ministry of Education of the People’s Republic of China, key results of National Education Statistics in 2021, 2022). In China, students with lower middle school examination scores typically attend vocational high schools. These students often face heightened pressure from schools, families, and society due to their academic performance, making them particularly vulnerable to mental health challenges. Compared to their peers in regular high schools, vocational students are more likely to exhibit emotional and behavioral issues stemming from differences in parenting styles, academic demands, and socio-cultural factors (Liu et al., 2022; Zheng et al., 2022). Furthermore, vocational school students report higher rates of smoking, drinking, fighting, running away from home, excessive internet use, internet addiction, and sexual activity (Zhang, 2013).

Therefore, the mental health challenges faced by vocational high school students, as a distinct group, warrant serious attention. This study aims to explore the prevalence of mental health problems among vocational high school students with varying levels of internet addiction in Henan Province, China, and to identify the factors influencing these issues. The findings are intended to inform the development of targeted mental health resources and support services for schools and communities, promoting the comprehensive development and well-being of these students.

## 2 Materials and methods

### 2.1 Sample and setting

From June to August 2018, an epidemiological survey was conducted to investigate the prevalence of mental health problems and associated factors among vocational high school students in Henan Province, China. At the time of the survey, over 2 million adolescents were enrolled in 852 high schools in the province, with an average of 2,465 students per school. Demographic data indicated that the number of students in regular high schools was approximately five times that of vocational high schools. Based on this proportion, the target sample size was set at 2,800 vocational high school students, representing one-fifth of the total population. Assuming 100 students would be sampled from each school (33 per grade), 28 schools were required.

A stratified cluster sampling method was employed to select participants from 28 vocational high schools across 17 provincial cities. Within each selected school, two to three classes per grade were randomly chosen based on class size. Class teachers invited all students in the selected classes to participate in the study, providing them with detailed written instructions and information about the data collection process. Participants completed the questionnaire during self-study periods under the supervision of the principal investigator.

### 2.2 Ethical considerations

This study was approved by the Ethics Committee of the Health Science Center at Xi’an Jiaotong University and the local schools participating in the study (Project No: 2018-296). All participants and their parents were fully informed about the study, its potential risks and benefits, and the role of participants, and they provided informed consent before data collection. Privacy and voluntary participation were ensured.

### 2.3 Instrumentation

#### 2.3.1 Demographic information

The demographic data were designed by the researchers based on a review of literature related to factors influencing the mental health of vocational high school students. The data encompassed four key dimensions: (1) Demographic Characteristics: Age, gender, ethnicity, grade, and permanent residence. (2) Lifestyle Factors: Boarding status, smoking, drinking, sexual behavior, physical illnesses, and chronic constipation. (3) Unhealthy Behaviors: Smoking, alcohol consumption, sexual activities, and gaming addiction. (4) Family Environment: Levels of loneliness and parents’ education.

#### 2.3.2 Mental health inventory of middle-school students

The Chinese version of the Middle School Mental Health Inventory (MMHI-60) scale was used to access the general mental health of students. It was developed by Wang et al. (2021) following a two-year longitudinal study of mental health problems among middle school students across more than 100 schools and has been successfully applied to middle school students in China (Zhao and Liao, 2016). The MMHI-60 consists of 60 items, each requiring respondents to rate on a five-point scale their recent experiences of specific symptoms or behaviors. The MMHI-60 includes 10 subscales reflecting the most relevant aspects of mental health: compulsiveness, paranoia, hostility, interpersonal sensitivity, depression, anxiety, academic pressure, poor adaptation, emotional disorders, and psychological imbalance. Scores are calculated by adding up the 60 items and dividing by 60, with each subscale score obtained by adding six items and dividing by six, producing subscale scores and an overall score ranging from 1 to 5. Higher scores indicate poorer mental health. According to Coledam et al. (2022), a cutoff score of 2 is associated with mental health problems, demonstrating good sensitivity and specificity in previous studies. The internal consistency reported by Wang et al. is considered adequate (test–retest reliability: 0.716–0.873; split-half reliability: 0.634–0.873). This scale has been authorised by the original author.

#### 2.3.3 Internet addiction test (IAT)

The Internet Addiction Test (IAT) comprises 20 items scored on a Likert scale from 1 (rarely) to 5 (always) (Young, 1998). Developed based on the pathological gambling criteria outlined in DSM-IV-TR (American Psychiatric Association, 2013), the IAT evaluates the focus and compulsion of internet use and its impact on daily life. According to Young (1998), respondents scoring ≥70 are categorized as addicted internet users. Scores between 40 and 69 indicate problematic internet use associated with general life difficulties, while scores ≤39 denote average internet users with minor control issues. This study used the Chinese version of the Internet Addiction Test (IAT) scale, which has been translated according to standard procedures. Previous studies have demonstrated high reliability for the Chinese version of IAT among adolescents, with Cronbach’s alpha values exceeding 0.80 (Wang et al., 2011). This scale has been authorised by the original author.

### 2.4 Data analysis

All survey data were recorded using EpiData 3.1 software and independently double-checked by two researchers to ensure accuracy. Statistical analyses were conducted using R software (version 1.3.1093). Continuous variables were presented as means with standard deviations (SD), while categorical variables were expressed as frequencies and percentages (*n*%). The prevalence of mental health problems was estimated for the general population and subgroups stratified by IAT scores, with 95% confidence intervals.

Univariate logistic regression was employed to identify potential factors associated with mental health problems. Variables significantly associated with mental health problems in univariate analysis, along with those highlighted in previous studies, were included as independent variables in the multivariate logistic regression analysis. Statistical significance was set at *p* < 0.05.

## 3 Results

### 3.1 Demographic information

A total of 3,051 students were recruited for this survey. Their age range was 14 to 20 years. Middle adolescence, i.e., 14–16 years old accounted for (31.0%) and late adolescence, i.e., 17–21 years old accounted for (69%). About half were male (51.3%) and from urban areas (64.4%). Most were Han Chinese (98.1%), lived in schools (74.9%) and came from two-parent families (91.8%). In total, 10.8% of the participants smoked, 18.6% drank alcohol, 5.1% had sexual intercourse, and 4.4% had physical illnesses. The majority of the students’ fathers (2,346; 76.9%) and mothers (2,458; 80.6%) had an educational level lower than that of the specialty. The characteristics of the participants are shown in Table 1.

**Table 1:** General information of the participants (*N* = 3,051).

	Total (*N* = 3,051)	Average user (*N* = 1825)	Problematic user (*N* = 958)	Addictive user (*N* = 268)	*p*-value
Gender					
Male	1,565 (51.3%)	745 (40.8%)	615 (64.2%)	205 (76.5%)	<0.001
Female	1,486 (48.7%)	1,080 (59.2%)	343 (35.8%)	63 (23.5%)	
Age					
14–16	1,191 (39.0%)	668 (36.6%)	404 (42.2%)	119 (44.4%)	0.008
17–21	1860 (61.0%)	1,157 (63.4%)	554 (57.8%)	149 (55.6%)	
Ethnicity					
Han	2,994 (98.1%)	1792 (98.2%)	944 (98.5%)	258 (96.3%)	0.113
Minority	57 (1.9%)	33 (1.8%)	14 (1.5%)	10 (3.7%)	
Inhabitation					
Rural	1965 (64.4%)	1,202 (65.9%)	636 (66.4%)	127 (47.4%)	<0.001
Urban	110 (3.6%)	53 (2.9%)	41 (4.3%)	16 (6.0%)	
Rural–urban continuum	976 (32.0%)	570 (31.2%)	281 (29.3%)	125 (46.6%)	
Grade					
First year	1,262 (41.4%)	742 (40.7%)	409 (42.7%)	111 (41.4%)	0.006
Second year	1,180 (38.7%)	678 (37.2%)	381 (39.8%)	121 (45.1%)	
Third year	609 (20.0%)	405 (22.2%)	168 (17.5%)	36 (13.4%)	
Residence on campus					
No	766 (25.1%)	517 (28.3%)	183 (19.1%)	66 (24.6%)	<0.001
Yes	2,285 (74.9%)	1,308 (71.7%)	775 (80.9%)	202 (75.4%)	
Smoking					
No	2,722 (89.2%)	1715 (94.0%)	840 (87.7%)	167 (62.3%)	<0.001
Yes	329 (10.8%)	110 (6.0%)	118 (12.3%)	101 (37.7%)	
Alcohol consumption					
No	2,484 (81.4%)	1,623 (88.9%)	726 (75.8%)	135 (50.4%)	<0.001
Yes	567 (18.6%)	202 (11.1%)	232 (24.2%)	133 (49.6%)	
Sexual activity					
No	2,894 (94.9%)	1753 (96.1%)	906 (94.6%)	235 (87.7%)	<0.001
Yes	157 (5.1%)	72 (3.9%)	52 (5.4%)	33 (12.3%)	
Physical disease					
No	2,916 (95.6%)	1760 (96.4%)	916 (95.6%)	240 (89.6%)	<0.001
Yes	135 (4.4%)	65 (3.6%)	42 (4.4%)	28 (10.4%)	
Chronic constipation					
No	2,768 (90.7%)	1,684 (92.3%)	863 (90.1%)	221 (82.5%)	<0.001
Yes	283 (9.3%)	141 (7.7%)	95 (9.9%)	47 (17.5%)	
Loneliness					
Never	1,074 (35.2%)	755 (41.4%)	254 (26.5%)	65 (24.3%)	<0.001
Mild	1,356 (44.4%)	823 (45.1%)	470 (49.1%)	63 (23.5%)	
Moderate	418 (13.7%)	191 (10.5%)	166 (17.3%)	61 (22.8%)	
Severe	113 (3.7%)	32 (1.8%)	50 (5.2%)	31 (11.6%)	
Very severe	90 (2.9%)	24 (1.3%)	18 (1.9%)	48 (17.9%)	
Single parent					
No	2,800 (91.8%)	1,693 (92.8%)	870 (90.8%)	237 (88.4%)	0.057
Yes	251 (8.2%)	132 (7.2%)	88 (9.2%)	31 (11.6%)	
Paternal education					
High school and below	2,346 (76.9%)	1,386 (75.9%)	761 (79.4%)	199 (74.3%)	<0.001
Junior college	396 (13.0%)	248 (13.6%)	129 (13.5%)	19 (7.1%)	
Bachelor	230 (7.5%)	157 (8.6%)	49 (5.1%)	24 (9.0%)	
Master	22 (0.7%)	10 (0.5%)	7 (0.7%)	5 (1.9%)	
Ph.D.	57 (1.9%)	24 (1.3%)	12 (1.3%)	21 (7.8%)	
Maternal education					
High school and below	2,458 (80.6%)	1,478 (81.0%)	780 (81.4%)	200 (74.6%)	<0.001
Junior college	322 (10.6%)	196 (10.7%)	104 (10.9%)	22 (8.2%)	
Bachelor	190 (6.2%)	115 (6.3%)	54 (5.6%)	21 (7.8%)	
Master	27 (0.9%)	10 (0.5%)	8 (0.8%)	9 (3.4%)	
Ph.D.	54 (1.8%)	26 (1.4%)	12 (1.3%)	16 (6.0%)	

### 3.2 Positive rate of mental health problems and univariate analysis of factors

The positivity rate of mental health problems among vocational high school students was 59% (95% CI = 57–61%), and the positivity rate of the average user group, problematic user group addictive user group was 65% (95% CI = 63–67%), 54% (95% CI = 51–57%), and 34% (95%CI = 28–40%).

Table 2 shows univariate analyses of demographic data, physical illness, lifestyle, and family factors associated with mental health problems. For the total population and the three groups, ethnic minorities, having a physical illness, chronic constipation, and loneliness were common influences on the mental health of vocational high school students (*p* < 0.05). In particular, for the total population, age, living in a city, living in a school, smoking or drinking alcohol, having sexual behavior, and being a single parent were associated with a high risk of mental health problems (*p* < 0.05). For average users, age, living in school, having sex, were associated with higher odds of mental health problems (*p* < 0.05)For problematic users, living in the city, smoking or drinking alcohol, having sex, were associated with higher risk of mental health problems (*p* < 0.05). For addictive users, age, second and third graders were associated with a higher risk of mental health problems (*p* < 0.05) (see Table 3).

**Table 2:** Univariate analysis of factors associated with mental health problems.

Positive rate	General users(*N* = 1825)			Problematic users(*N* = 958)		Addictive user(*N* = 268)		Total(*N* = 3051)
	Positive rate	OR	Positive rate	OR		Positive rate		OR	Positive rate	OR
Total	0.65 (0.63–0.67)		0.54 (0.51–0.57)		0.34 (0.28–0.4)		0.59 (0.57–0.61)	
Gender								
Male	0.34 (0.3–0.37)		0.46 (0.42–0.5)		0.63 (0.56–0.7)		0.42 (0.4–0.45)	
Female	0.35 (0.33–0.38)	1.08 (0.89–1.31)	0.45 (0.4–0.51)	0.98 (0.75–1.28)	0.75 (0.62–0.84)	1.69 (0.91–3.27)	0.39 (0.37–0.42)	0.88 (0.76–1.02)
Age								
14–16	0.31 (0.28–0.35)		0.44 (0.40–0.50)		0.71 (0.63–0.78)		0.39 (0.36–0.41)	
17–21	0.37 (0.34–0.40)	1.28 (1.05–1.57)	0.47 (0.42–0.51)	1.07 (0.83–1.39)	0.59 (0.50–0.68)	1.67 (1.00–2.78)	0.42 (0.40–0.45)	1.17 (1.01–1.36)
Ethnicity								
Han	0.34 (0.32–0.37)		0.45 (0.42–0.49)		0.67 (0.61–0.72)		0.41 (0.39–0.42)	
Minority	0.52 (0.34–0.69)	2.03 (1.01–4.08)	0.79 (0.49–0.94)	4.42 (1.37–19.64)	0.5 (0.24–0.76)	0.5 (0.14–1.84)	0.58 (0.44–0.71)	2.01 (1.19–3.45)
Inhabitation								
Rural	0.4 (0.37–0.43)		0.44 (0.4–0.48)		0.64 (0.55–0.72)		0.43 (0.41–0.45)	
Urban	0.42 (0.28–0.56)	1.06 (0.6–1.84)	0.76 (0.59–0.87)	3.94 (1.97–8.6)	0.63 (0.36–0.84)	0.95 (0.33–2.94)	0.57 (0.47–0.67)	1.78 (1.21–2.64)
Rural–urban continuum	0.22 (0.19–0.26)	0.43 (0.34–0.54)	0.46 (0.4–0.52)	1.06 (0.8–1.41)	0.69 (0.6–0.77)	1.25 (0.74–2.12)	0.35 (0.32–0.38)	0.72 (0.61–0.84)
Grade								
First year	0.35 (0.32–0.39)		0.45 (0.4–0.5)		0.55 (0.45–0.64)		0.4 (0.37–0.43)	
Second year	0.37 (0.33–0.4)	1.05 (0.85–1.3)	0.47 (0.42–0.52)	1.11 (0.84–1.46)	0.74 (0.65–0.81)	2.28 (1.32–3.98)	0.44 (0.41–0.47)	1.16 (0.99–1.36)
Third year	0.3 (0.26–0.35)	0.79 (0.6–1.02)	0.45 (0.38–0.53)	1.02 (0.71–1.46)	0.75 (0.57–0.87)	2.46 (1.09–5.98)	0.37 (0.33–0.41)	0.87 (0.71–1.06)
Residence on campus								
No	0.26 (0.22–0.3)		0.48 (0.41–0.56)		0.71 (0.59–0.81)		0.35 (0.32–0.38)	
Yes	0.38 (0.36–0.41)	1.81 (1.45–2.28)	0.45 (0.42–0.49)	0.89 (0.65–1.23)	0.64 (0.57–0.71)	0.73 (0.39–1.32)	0.43 (0.41–0.45)	1.41 (1.19–1.67)
Smoking								
No	0.34 (0.32–0.37)		0.43 (0.4–0.47)		0.63 (0.55–0.7)		0.39 (0.37–0.41)	
Yes	0.4 (0.31–0.5)	1.27 (0.85–1.88)	0.65 (0.56–0.74)	2.48 (1.67–3.74)	0.71 (0.61–0.8)	1.47 (0.87–2.52)	0.59 (0.53–0.64)	2.24 (1.78–2.83)
Alcohol consumption								
No	0.34 (0.32–0.36)		0.44 (0.4–0.47)		0.62 (0.53–0.7)		0.38 (0.36–0.4)	
Yes	0.42 (0.35–0.49)	1.39 (1.03–1.87)	0.53 (0.46–0.59)	1.43 (1.06–1.93)	0.7 (0.61–0.77)	1.41 (0.85–2.35)	0.53 (0.49–0.57)	1.8 (1.5–2.17)
Sexual activity								
No	0.34 (0.32–0.36)		0.45 (0.42–0.48)		0.66 (0.59–0.72)		0.4 (0.38–0.42)	
Yes	0.47 (0.35–0.59)	1.72 (1.07–2.77)	0.62 (0.47–0.74)	1.96 (1.11–3.53)	0.67 (0.48–0.81)	1.03 (0.49–2.31)	0.56 (0.48–0.64)	1.9 (1.38–2.64)
Physical disease								
No	0.34 (0.32–0.36)		0.45 (0.42–0.48)		0.64 (0.57–0.7)		0.4 (0.38–0.42)	
Yes	0.54 (0.41–0.66)	2.27 (1.38–3.75)	0.64 (0.48–0.78)	2.2 (1.17–4.29)	0.86 (0.66–0.95)	3.41 (1.27–11.89)	0.64 (0.55–0.72)	2.65 (1.86–3.81)
Chronic constipation								
No	0.33 (0.31–0.35)		0.44 (0.41–0.47)		0.62 (0.55–0.68)		0.39 (0.37–0.41)	
Yes	0.53 (0.45–0.62)	2.29 (1.62–3.25)	0.62 (0.52–0.72)	2.08 (1.35–3.25)	0.85 (0.71–0.93)	3.5 (1.59–8.87)	0.61 (0.56–0.67)	2.51 (1.96–3.24)
Loneliness								
Never	0.14 (0.12–0.17)		0.22 (0.17–0.27)		0.35 (0.24–0.48)		0.17 (0.15–0.2)	
Mild	0.42 (0.39–0.46)	4.44 (3.48–5.71)	0.45 (0.4–0.5)	2.95 (2.09–4.21)	0.57 (0.44–0.69)	2.43 (1.2–5.02)	0.44 (0.41–0.46)	3.76 (3.11–4.56)
Moderate	0.69 (0.61–0.75)	13.37 (9.3–19.43)	0.68 (0.6–0.75)	7.71 (4.99–12.1)	0.77 (0.64–0.86)	6.13 (2.86–13.8)	0.7 (0.65–0.74)	11.08 (8.55–14.45)
Severe	0.91 (0.74–0.98)	59.19 (20.57–250.24)	0.86 (0.73–0.94)	22.23 (10.04–56.56)	0.94 (0.77–0.99)	26.48 (7.11–173.28)	0.89 (0.82–0.94)	40.71 (22.8–79.48)
Very severe	0.88 (0.67–0.97)	42.86 (14.46–183.71)	0.94 (0.71–1)	61.51 (12.22–1120.57)	0.88 (0.74–0.95)	12.78 (5.02–37.67)	0.89 (0.8–0.94)	38.7 (20.62–80.87)
Single parent								
No	0.34 (0.32–0.37)		0.45 (0.42–0.49)		0.65 (0.59–0.71)		0.4 (0.38–0.42)	
Yes	0.41 (0.33–0.5)	1.33 (0.92–1.91)	0.51 (0.4–0.62)	1.26 (0.81–1.96)	0.71 (0.52–0.85)	1.29 (0.59–3.08)	0.48 (0.42–0.55)	1.38 (1.06–1.79)
Paternal education								
High school and below	0.39 (0.36–0.41)		0.47 (0.43–0.51)		0.67 (0.6–0.73)		0.44 (0.42–0.46)	
Junior college	0.19 (0.14–0.24)	0.36 (0.25–0.5)	0.39 (0.3–0.48)	0.71 (0.48–1.04)	0.58 (0.34–0.79)	0.68 (0.26–1.84)	0.27 (0.23–0.32)	0.47 (0.37–0.6)
Bachelor	0.24 (0.18–0.32)	0.5 (0.34–0.73)	0.41 (0.27–0.56)	0.78 (0.43–1.39)	0.71 (0.49–0.87)	1.21 (0.49–3.25)	0.33 (0.27–0.39)	0.62 (0.46–0.82)
Master	0.2 (0.04–0.56)	0.39 (0.06–1.58)	0.57 (0.2–0.88)	1.5 (0.33–7.66)	0.8 (0.3–0.99)	1.98 (0.29–39.24)	0.45 (0.25–0.67)	1.07 (0.45–2.48)
Ph.D.	0.38 (0.2–0.59)	0.95 (0.39–2.14)	0.58 (0.29–0.84)	1.58 (0.5–5.37)	0.57 (0.34–0.77)	0.66 (0.27–1.7)	0.49 (0.36–0.63)	1.24 (0.73–2.09)
Maternal education								
High school and below	0.37 (0.35–0.4)		0.46 (0.43–0.5)		0.66 (0.58–0.72)		0.42 (0.41–0.44)	
Junior college	0.19 (0.14–0.25)	0.39 (0.27–0.56)	0.43 (0.34–0.53)	0.89 (0.58–1.33)	0.68 (0.45–0.85)	1.13 (0.45–3.08)	0.3 (0.25–0.36)	0.58 (0.45–0.75)
Bachelor	0.27 (0.19–0.36)	0.62 (0.4–0.94)	0.41 (0.28–0.55)	0.8 (0.45–1.39)	0.67 (0.43–0.85)	1.05 (0.42–2.89)	0.35 (0.29–0.43)	0.74 (0.54–1)
Master	0.3 (0.08–0.65)	0.72 (0.15–2.6)	0.38 (0.1–0.74)	0.7(0.14–2.86)	0.67(0.31–0.91)	1.05(0.27–5.11)	0.44(0.26–0.64)	1.08(0.5–2.32)
Ph.D.	0.38 (0.21–0.59)	1.05 (0.46–2.29)	0.67 (0.35–0.89)	2.32 (0.72–8.76)	0.69 (0.41–0.88)	1.16 (0.4–3.8)	0.54 (0.4–0.67)	1.57 (0.92–2.72)

**Table 3:** Multivariable analysis of factors associated with mental health problems (OR (95% CI)).

	Total (*N* = 3,051)	Average user	Problematic user	Addictive user
		(*N* = 1825)	(*N* = 958)	(*N* = 268)
Age				
14–16 (Ref)				
17–21	1.16 (0.98–1.38)			
Ethnicity				
Han (Ref)				
Minority	1.95 (1.05–3.66)	1.98 (1.08–3.65)	1.99 (1.09–3.65)	
Inhabitation				
Rural (Ref)				
Urban	1.43 (0.88–2.32)	1.36 (0.85–2.19)	1.20 (0.76–1.91)	
Rural–urban continuum	0.76 (0.62–0.95)	0.76 (0.61–0.93)	0.61 (0.51–0.74)	
Grade				
First year (Ref)				
Second year				1.04 (0.87–1.25)
Third year				0.73 (0.59–0.92)
Residence on campus				
No (Ref)				
Yes	1.15 (0.91–1.44)	1.13 (0.91–1.41)		
Smoking				
No (Ref)				
Yes	1.51 (1.05–2.16)		1.51 (1.07–2.14)	
Alcohol consumption				
No (Ref)				
Yes	1.01 (0.77–1.33)	1.14 (0.9–1.42)	1.02 (0.79–1.33)	
Sexual activity				
No (Ref)				
Yes	1.33 (0.57–3.19)	1.49 (1.01–2.21)	1.28 (0.86–1.92)	
Physical disease				
No (Ref)				
Yes	1.45 (0.96–2.22)	1.44 (0.96–2.18)	1.39 (0.92–2.1)	1.55 (1.03–2.33)
Chronic constipation				
No (Ref)				
Yes	1.88 (1.4–2.54)	1.99 (1.49–2.66)	1.88 (1.41–2.5)	1.96 (1.48–2.61)
Loneliness				
Never (Ref)				
Mild	3.61 (2.96–4.41)	3.62 (2.98–4.41)	3.63 (2.99–4.42)	3.76 (3.1–4.57)
Moderate	10.39 (7.93–13.7)	10.05 (7.7–13.18)	10.22 (7.84–13.38)	10.56 (8.13–13.79)
Severe	37.64 (20.76–74.35)	37.81 (20.95–74.42)	37.34 (20.74–73.36)	39.05 (21.78–76.47)
Very severe	32.78 (16.97–70.03)	32.95 (17.17–70.03)	31.7 (16.59–67.12)	33.63 (17.80–70.62)
Single parent				
No (Ref)				
Yes	1.02 (0.74–1.41)			
Paternal education				
High school and below (Ref)				
Junior college	0.53 (0.38–0.74)	0.55 (0.4–0.76)		
Bachelor	0.52 (0.33–0.82)	0.52 (0.33–0.81)		
Master	0.8 (0.24–2.49)	0.76 (0.23–2.31)		
Ph.D.	0.23 (0.04–1.22)	0.2 (0.03–1.02)		
Maternal education				
High school and below (Ref)				
Junior college	1.02 (0.71–1.45)	1.02 (0.72–1.45)		
Bachelor	1.43 (0.88–2.3)	1.41 (0.88–2.24)		
Master	1.45 (0.51–4.24)	1.65 (0.59–4.65)		
Ph.D.	6.36 (1.15–42.08)	7.3 (1.39–45.93)		

### 3.3 Factors associated with mental health problems

In the total population, ethnic minority, smoking, having sexual behavior, physical illness, chronic constipation, feeling lonely, and mother’s education were factors associated with higher odds of mental health problems (*p* < 0.05). In the average user group, ethnic minority, smoking, having sexual intercourse, feeling lonely, constipation, and mother’s education were factors associated with higher odds of mental health problems (*p* < 0.05). In the problematic user group, ethnic minority, sexual behavior, constipation, loneliness, and mother’s education were factors associated with higher odds of mental health problems (*p* < 0.05). In the ADDICTIVE USER group, physical illness, chronic constipation, and loneliness were factors associated with higher odds of mental health problems (p < 0.05).

## 4 Discussion

The study results show that the prevalence of mental health issues among vocational high school students is 59% (95% CI = 57–61%). For the average user group, the problematic user group, and the addictive user group, the prevalence rates are 65% (95% CI = 63–67%), 54% (95% CI = 51–57%), and 34% (95% CI = 28–40%), respectively. Loneliness, ethnicity, maternal education level, smoking, sexual behavior, constipation, and physical illness are the main influencing factors, with some differences observed among the general population and different user groups.

Additionally, a cross-sectional study of 675 Brazilian high school students revealed higher mental health symptom prevalence in vocational schools than in regular high schools (Coledam et al., 2022). The lifetime prevalence of D-SIB in vocational schools was significantly higher compared to regular high schools [*X*^2^(1) = 12.231, *p* < 0.001]. Comparison of the mental health problems of vocational high school students in China with those in other countries reveals both similarities and differences in prevalence, risk factors and demographics. For example, the study in Hunan Province found a high prevalence of insomnia, anxiety and depression, and a significant association between these conditions and eating disorders. However, socio-demographic factors such as family background and parental education level had little correlation with these health problems (Liu et al., 2022). In contrast, in the United States, where more than one-third of high school students reported poor mental health during the COVID-19 pandemic, school connectivity and virtual interactions had a significant impact on mental health outcomes. This suggests that a sense of connectedness to school and others plays a protective role against mental health problems (Jones et al., 2022).

In another study conducted in Henan Province, China, 41.8% of high school students reported mental health problems, with significant risk factors including senior grades, physical illness, chronic constipation, drinking, sexual behavior, campus residency, living in non-urban areas, or coming from single-parent families (Luo et al., 2020). The differences in survey results between different countries and regions can be attributed to several factors, including cultural differences, variations in school and social environments, and differences in research methodologies. For example, studies in the U.S. emphasize the connectivity and virtual interaction of schools, reflecting the country’s focus on the social aspects of mental health, while studies in China emphasize behavioral and demographic factors. Additionally, U.S. studies have explicitly discussed the impact of the COVID-19 pandemic on mental health, highlighting the influence of external stressors and the importance of social support systems. Overall, these studies indicate that while high school students face common global trends in mental health problems, local cultural, social, and environmental factors significantly influence specific risk factors and prevalence rates in different regions.

The results of this study show that loneliness is the strongest correlate of mental health problems in all populations. The internet is a part of everyday life, primarily used for communication and social interaction. However, its excessive use may lead to dependency on social networks. Loneliness is a significant factor in adolescent mental health, often associated with mood disorders, anxiety, depression, and even suicidal behavior, potentially linked to a lack of social networks or inadequate social skills. Hobson (1974) revealed that feelings of isolation or social exclusion can impair cognitive functions, willpower, and the immune system, severely affecting both physical and mental health. Matthews et al. (2016) from a behavioral genetics perspective, found significant correlations between social isolation, loneliness, and symptoms of depression among adolescents. The study showed that social isolation and loneliness are significantly related to symptoms of depression. Furthermore, the links between these psychosocial traits can largely be explained by common genetic factors. Persistent loneliness can lead adolescents to develop a negative self-perception, which can further lower self-esteem and increase the risk of depression symptoms. Firstly, adolescence is a critical period for the development of social and emotional skills, and social interaction is crucial for their personal growth and emotional stability. A lack of friendship and social support can make adolescents feel isolated, increasing their stress and anxiety levels. Secondly, adolescence is an important stage for developing self-identity. Persistent loneliness can interfere with this process, hindering identity development. This not only affects adolescents’ mental health but may also impact their long-term personality formation and social functions.

The results of this study show that ethnic minorities are influential factors in the emergence of mental health problems among vocational high school students in the total population, average user group and problematic user group. Ethnic minority students may face additional psychological stress due to cultural differences, language barriers, lower socio-economic status, and experiences of discrimination or isolation, all of which can impact their mental health. Families of ethnic minorities might face more economic hardships and social marginalization, indirectly affecting adolescents. Social discrimination and a sense of isolation can also be significant sources of psychological stress, particularly prevalent among ethnic minority groups (Letourneau et al., 2013). Moreover, mothers from different cultural backgrounds might have varying parenting behaviors and expectations. For instance, some cultures might emphasize authority and obedience more, which could limit adolescents’ self-expression and independence, thereby potentially impacting their mental health (Dune et al., 2018; Sahithya et al., 2019).

The results of this study found that maternal education was an influential factor in the emergence of mental health problems among vocational high school students in both the total population and average user groups. The higher educational level of the mother may be related to family expectations and academic pressure. Studies indicate that family and social factors significantly impact adolescents’ mental health. Systematic reviews of interactive interventions in schools and communities highlight the importance of involving students, teachers, families, and mental health professionals. These interventions are linked to improvements in children and adolescents’ social skills, personal well-being, and reductions in destructive behaviors and emotional symptoms such as depression and anxiety (García-Carrión et al., 2019; Wang et al., 2021). Further explored the bidirectional relationship between family function and adolescent depressive symptoms, indicating both family-driven and child-driven influences. The study underscores the necessity of considering the dynamic interactions between family function and adolescent mental health, particularly emphasizing the significant role of the family in adolescent development within the Chinese context.

The results of this study show that smoking is an influential factor affecting the emergence of mental health problems among vocational high school students in the total population and problematic user group. Research shows that a significant proportion of students in vocational education engage in smoking, at higher rates than those in non-vocational education (Corcoran and Allegrante, 1989). For instance, a study by Lu et al. (2023) found that smoking and drinking are more prevalent among students engaging in non-suicidal self-injury (NSSI), suggesting a correlation between these risky behaviors and mental health problems. Additionally, the incidence of Non-Suicidal Self-Injury (NSSI) is significantly higher among students who consume alcohol, suggesting that drinking may exacerbate mental health problems. Smoking has been causally linked to depressive symptoms among adolescent female (Lien et al., 2009). Smokers are 1.5–2.0 times more likely to suffer from depression than non-smokers (Steuber and Danner, 2006). These studies collectively illustrate how smoking impacts various aspects of adolescent mental health and emphasize the importance of early prevention and intervention.

In the problematic user group, sexual behavior is a factor affecting mental health problems among vocational high school students. Recent research in the United States has found a negative correlation between casual sexual behavior and mental health (Bersamin et al., 2014). Additionally, a 2006 U.S. study found that women engaging in casual sexual activities exhibited more symptoms of depression compared to their male counterparts (Grello et al., 2006). A study from Rwanda also supports this conclusion, linking risk-taking behaviors (RSB) with mental health challenges (Ndagijimana et al., 2023). Among Chinese adolescents, clustered health-risk behaviors, including smoking and drinking, are significantly associated with mental health problems. Therefore, addressing mental health problems is crucial for safer sexual behavior.

In the addictive user group, physical illness is a factor affecting mental health problems in vocational high school students. Chronic constipation typically causes physical discomfort and pain, which can lead to missed school days and social withdrawal. The stress of managing a chronic illness like constipation can cause psychological burdens. Adolescents may become more irritable and emotionally low due to persistent discomfort and frustration related to managing their conditions. Similarly, issues like chronic constipation can affect adolescents’ body image and self-esteem, particularly if they feel less control over their bodily functions compared to their peers (Yamada et al., 2021). Evidence suggests that adolescents with chronic constipation may have higher rates of psychological disorders, such as depression and anxiety. Disorders like anorexia nervosa also have a higher prevalence among those with constipation, indicating a complex internal relationship.

Students with physical illnesses may be more susceptible to mental health problems due to physical discomfort, stress from treatment, or concerns about their illness. Research shows that adolescents with physical conditions like asthma or epilepsy are at a higher risk of psychological distress compared to their healthy peers (Ferro, 2014). Additionally, adolescent mental and physical health issues are interrelated, with mental disorders potentially exacerbating physical diseases and vice versa, affecting their overall quality of life. Physical illnesses can lead to a higher incidence of mental health problems for several reasons: Chronic stress and pain: Physical diseases often cause chronic pain and stress, which can lead to the development of mental health problems such as depression and anxiety. Impact on Daily Functioning and Quality of Life: Physical health problems can restrict students’ ability to participate in activities, negatively affecting their social lives and academic performance, leading to feelings of isolation and decreased self-esteem. Medication Side Effects: Some medications used to treat physical illnesses may have side effects that impact mental health, causing mood swings, depression, or anxiety.

### 4.1 Limitations

This cross-sectional study cannot establish causality between mental health problems and their influencing factors. Longitudinal studies and randomized controlled trials should be conducted to identify risk factors and formulate corresponding interventions.

Additionally, as all samples were from Henan, China, this limits the generalizability of the results to the national population of high school students. The sample size for this study was primarily determined based on provincial enrolment ratios. Future studies will calculate sample sizes based on previous prevalence rates to enhance methodological rigour and should involve a larger, multi-center national sample to enhance the generalizability of the findings.

Finally, this study only assessed the prevalence and risk factors of internet addiction (IA) and its association with mental health problems. Future studies will use mixed research methods to explore the mental health challenges of problematic users in greater depth.

## 5 Conclusion

The study found a high prevalence of mental health problems among vocational high school students, with loneliness, ethnicity, maternal education, smoking, sexual behavior, constipation, and physical illness being the main influencing factors. These factors varied across the general population, average user groups, problematic user groups, and addictive user groups. This knowledge helps enhance our understanding of the risk factors related to the mental health of vocational high school students and paves the way for targeted intervention measures. Future research could involve large-scale randomized controlled trials to test the effectiveness of these interventions.

## Data Availability

The raw data supporting the conclusions of this article will be made available by the authors, without undue reservation.
